# The Bone Marrow Edema Links to an Osteoclastic Environment and Precedes Synovitis During the Development of Collagen Induced Arthritis

**DOI:** 10.3389/fimmu.2019.00884

**Published:** 2019-04-24

**Authors:** Fang Wang, Aishu Luo, Wenhua Xuan, Liang Qi, Qing Wu, Ke Gan, Qiande Zhang, Miaojia Zhang, Wenfeng Tan

**Affiliations:** ^1^Department of Cardiology, The First Affiliated Hospital of Nanjing Medical University, Nanjing, China; ^2^Department of Rheumatology, The First Affiliated Hospital of Nanjing Medical University, Nanjing, China; ^3^Department of Radiology, The First Affiliated Hospital of Nanjing Medical University, Nanjing, China; ^4^Department of Traditional Chinese Medicine, Nanjing Traditional Chinese Medicine University, Nanjing, China; ^5^Department of Chinese Medicine, Nanjing Medicine University Institute of Integration of Traditional Chinese and Western Medicine, Nanjing, China

**Keywords:** bone marrow edema, osteoclast, synovitis, bone marrow environment, collagen induced arthritis

## Abstract

**Objectives:** To determine the relationship between bone marrow edema (BME), synovitis, and bone erosion longitudinally using a collagen induced arthritis mice (CIA) model and to explore the potential pathogenic role of BME in bone erosion.

**Methods:** CIA was induced in DBA/1J mice. BME and corresponding clinical symptoms of arthritis and synovitis during the different time points of CIA development were assayed by magnetic resonance imaging (MRI), arthritis sore, and histologic analyses. The expression of osteoclasts (OCs), OCs-related cytokines, and immune cells in bone marrow were determined by flow cytometry, immunohistochemistry, immunofluorescence staining, and real-time PCR. The OCs formation was estimated using *in vitro* assays.

**Results:** MRI detected BME could emerge at day 25 in 70% mice after the first immunization (*n* = 10), when there were not any arthritic symptoms, histological or MRI synovitis. At day 28, BME occurred in 90% mice whereas the arthritic symptom and histological synovitis were only presented in 30 and 20% CIA mice at that time (*n* = 10). The emergence of BME was associated with an increased bone marrow OCs number and an altered distribution of OCs adherent to subchondral bone surface, which resulted in increased subchondral erosion and decreased trabecular bone number during the CIA process. Obvious marrow environment changes were identified after BME emergence, consisting of multiple OCs related signals, including highly expressed RANKL, increased proinflammatory cytokines and chemokines, and highly activated T cells and monocytes.

**Conclusions:** BME reflects a unique marrow “osteoclastic environment,” preceding the arthritic symptoms and synovitis during the development of CIA.

## Introduction

Bone erosion is a central pathogenic event and is associated with poor functional outcome in rheumatoid arthritis (RA). Synovitis is traditionally regarded as the primary trigger for bone erosion and fibroblast-like synoviocytes (FLS) play a central role in this process (1, 2). Compared with normal FLS, RA-FLS exhibits a unique aggressive and invasive property. These cells could excessively proliferate and produce a mass of pro-inflammatory cytokines, chemokines, and proteases that degrade the extracellular matrix, and invade into cartilage and bone (1). However, this synovitis-centered concept has been challenged by the bone-centered concept based on the finding of bone marrow edema (BME) in magnetic resonance imaging (MRI) in RA patients.

MRI-detected BME is initially considered as a sensitive marker of histological inflammation for early diagnosis of RA (3–6). The subsequent studies suggest that BME is closely associated with erosive progression in RA. BME at disease onset could predict progression of joint damage 1–6 years later (7–10). Although how the appearance of BME is associated with bone erosion remains unclear, these observations highlight that BME is an important part of RA pathology, perhaps directly representing the early event of erosions.

BME is also called osteitis, implying that adipose tissue is replaced by inflammatory cells infiltrating in bone marrow in the inflammatory condition (7). According to the synovitis-centered concept, BME is a consequence of synovitis invading into bone, resulting in the inflammation communication between the synovium tissue and bone marrow. If bone erosion is driven by synovitis, cartilage change that derived from inflammatory cytokines degradation bone matrix should be earlier than the bone change (11). However, MRI studies have noted that the occurrence of BME and erosions precede cartilage thinning in early RA (11). Most importantly, a longitudinal MRI study has recently found that the association of BME with erosive progression is independent of local synovitis (12). Some other studies have also demonstrated that synovitis and joint erosion could be uncoupled in RA. Bone erosion could be found in joints without clinical traits of synovitis (13). Moreover, a cohort study found that up to 40% of patients show an on-going progressive erosive disease despite DAS28 improvement or EULAR remission in these patients (14). Collectively, these findings imply that, in addition to synovitis, BME related pathway might participate in the bone erosion in early RA.

To date, the relationship between BME, synovitis, and bone erosion has not been thoroughly studied. This is because it is impossible to carry out invasive procedures in human subjects to study the MRI and the histographic appearances longitudinally. Using collagen induced mice arthritis model, we longitudinally observed time course of BME, synovitis, and bone erosion in a same joint during CIA development. Our data demonstrate that MRI BME precedes the synovitis and arthritic symptoms during the development of CIA. Moreover, we have found that BME reflects a unique marrow “osteoclastic environment” in the CIA process. These findings provide a new perspective for comprehending the development of erosions in RA.

## Methods

### Induction of Collagen-Induced Arthritis (CIA) in DBA/1J Mice

The induction of CIA was described previously (15). Briefly, male 6 week-old DBA/1J mice (Shanghai Laboratory Animal Center, Chinese Academy of Science) were injected intradermally at the base of the tail with 200 μg bovine CII (Chondrex, USA) emulsified with complete Freund adjuvant (CFA; Chondrex). At day 21, a booster immunization in the same manner was given to it. The animal experiments were performed in accordance with the guidelines approved by Institutional Animal Care and Use Committee of Nanjing Medical University.

### Clinical and Histological Assessment of CIA Mice

Signs of arthritis based on visual symptoms of four limbs were assessed every 2 or 3 days after the boost in DBA/1J mice. The symptoms were graded and scored as previously described (16). Briefly, all four limbs of the mice were evaluated and scored from 0 to 4 according to the following scale: 0 = no swelling; 1 = slight swelling and erythema confined to either ankle or mid foot; 2 = slight swelling extending from ankle to mid foot; 3 = moderate welling from ankle to metatarsal joints; 4 = severe swelling in the ankle, foot, and digits.

For histological assessment, the joint tissue was fixed overnight in 4% paraformaldehyde and decalcified using EDTA. The material was then embedded with paraffin and cut into 1–2 μm H&E staining. The histological arthritis score was determined in a blinded fashion for changes in synovial lining, cellular infiltrate, cartilage damage, and pannus as previously described (16).

To analyze osteoclast in joint tissues, each joint section was treated sequentially with anti-mouse TRAP antibody (Santa Cruz Biotechnology, Inc.) and the peroxidase conjugated goat anti-mouse IgG (H + L; Millipore). Sections were counter stained with hematoxylin (Sigma).

### Magnetic Resonance Imaging (MRI) and Data Analysis

Mice from days 20, 25, 28, 35, and 45 after the first immunization were used for MRI scanning, respectively (*n* = 10, at each time point). MRI studies were performed using a Bruker pharmscan 7.0T scanner (Bruker Biospin, Ettlingen, Germany) with 38 mm coil. Briefly, mice were anesthetized in 2% inhaled isoflurane in oxygen and then positioned on an imaging platform for localization scanning. The respiratory rate needed to maintain of 60–100 breaths/min. The scanning sequence included short time inversion recovery and T2 mapping scanning. The acquisition parameters were as follows: repetition time (TR) = 3,500 ms, echo time (TE) = 45 ms, matrix = 256 × 256, field of view = 2.2 × 1.8 mm, slice thickness = 0.5 mm, scanning time = 11 min, repeat 3 times. Each sequence scanning was completed after the emergence of eight images and then automatically generated T2 mapping graph.

The Bruker Biospin software was used for image analysis. To perform quantitative assessments of MRI signal intensities, region of interest (ROI) circles (1 mm^2^) was drawn on five contiguous 2-dimensional slices in the tibial marrow. The ROI was copied and placed in the gastrocnemius muscle. The muscle served as a normalization tissue, since its MRI signal with and without contrast was not affected by joint inflammation. The average value of mean marrow signal intensity divided by mean muscle signal intensity for five slices was known as normalized bone marrow intensity (NBMI).

### Flow Cytometric Analysis

The following flow cytometric antibodies were purchased from BioLegend (BioLegend, Cambridge, UK): CD4 (GK1.5), CD45R/B220 (RA3-6B2), CD14 (Sa14-2), CD138 (281-2), CD254(IK22/5), and CD11b(M1/70). Gr-1(RB6-8C5) was purchased from eBioscience Inc. (San Diego, CA). For flow cytometry, cells were isolated from bone marrow of CIA and control mice. About 1 × 10^6^ bone marrow cells were incubated with indicated antibody mixtures. In all staining, dead cells were excluded using an Aqua Live/Dead fixable staining reagent (Invitrogen), and doublets were excluded by FSC-A vs. FSC-H gating. Data were acquired using FACSCalibur flow cytometer (BD Biosciences) and analyzed using the Flowjo 7.6 software.

### Immunofluorescence Staining

Bone marrow cells were obtained at days 20, 25, 28, 35, and 45 mice after the first immunization. Cells were cultured at 37°C in 5% CO_2_ using α-MEM (Invitrogen, Grand Island, NY, USA) supplemented with 10% fetal bovine serum (FBS; Invitrogen) and penicillin/streptomycin (Invitrogen) about 5 days. Adherent cells were fixed in 4% paraformaldehyde for 20 min, and then permeabilized in 0.2% Triton X-100 and blocked with 3% BSA in PBS for 30 min at room temperature. All antibodies were diluted in 3% BSA in PBS. Primary Trap and RANKL antibodies were applied on samples overnight at 4°C. Secondary antibody FITC-donkey anti-rabbit IgG antibodies were added to samples for 1 h incubation at room temperature. Then DAPI (Vector Laboratories, H1200) was used to stain nuclei. After the staining process was completed, all samples were observed under Zeiss microscope and cells were quantified using image analysis program.

### Osteoclast Differentiation and TRAP Staining

Bone marrow cells from different time points were seeded into 6-well plates (5 × 10^4^ cells/well) and were treated with M-CSF (60 ng/ml) for 2 days (R&D Systems China Co. Ltd) and then were treated with RANKL (100 ng/ml) (PeproTech) for 5 days (change the media every 3 days). Then cells were washed twice with 1 × PBS, fixed for 10 min with 4% paraformaldehyde, and stained for TRAP using a leukocyte acid phosphatase cytochemistry kit according to the manufacturer's instructions. TRAP-positive multinucleated cells with three or more nuclei were counted as osteoclasts under a light microscope.

### RNA Extraction and Real-Time Quantitative PCR

Total RNA was extracted using TRIzol reagent (Invitrogen, Carlsbad, CA, USA) and cDNA was synthesized by the RNA PCR Core Kit (Applied Biosystems, Branchburg, NJ, USA). Levels of gene expression were quantified by SYBR Green real-time PCR using an ABI Prism 7900 Sequence Detection System. The sequences of the primers were listed in Table S1. Relative expression was calculated with normalization to GAPDH values by using the 2^ΔΔ^ Ct method.

### Statistical Analysis

Data were presented as mean ± SD. Statistics were performed using Kruskal-Wallis H test for multiple groups comparison. All statistical analyses were performed using GraphPad Prism 7.05 and *p* < 0.05 was considered statistically significant.

## Results

### BME Precedes the Arthritic Symptoms and Synovitis During the Development of CIA

**Figure 1:**
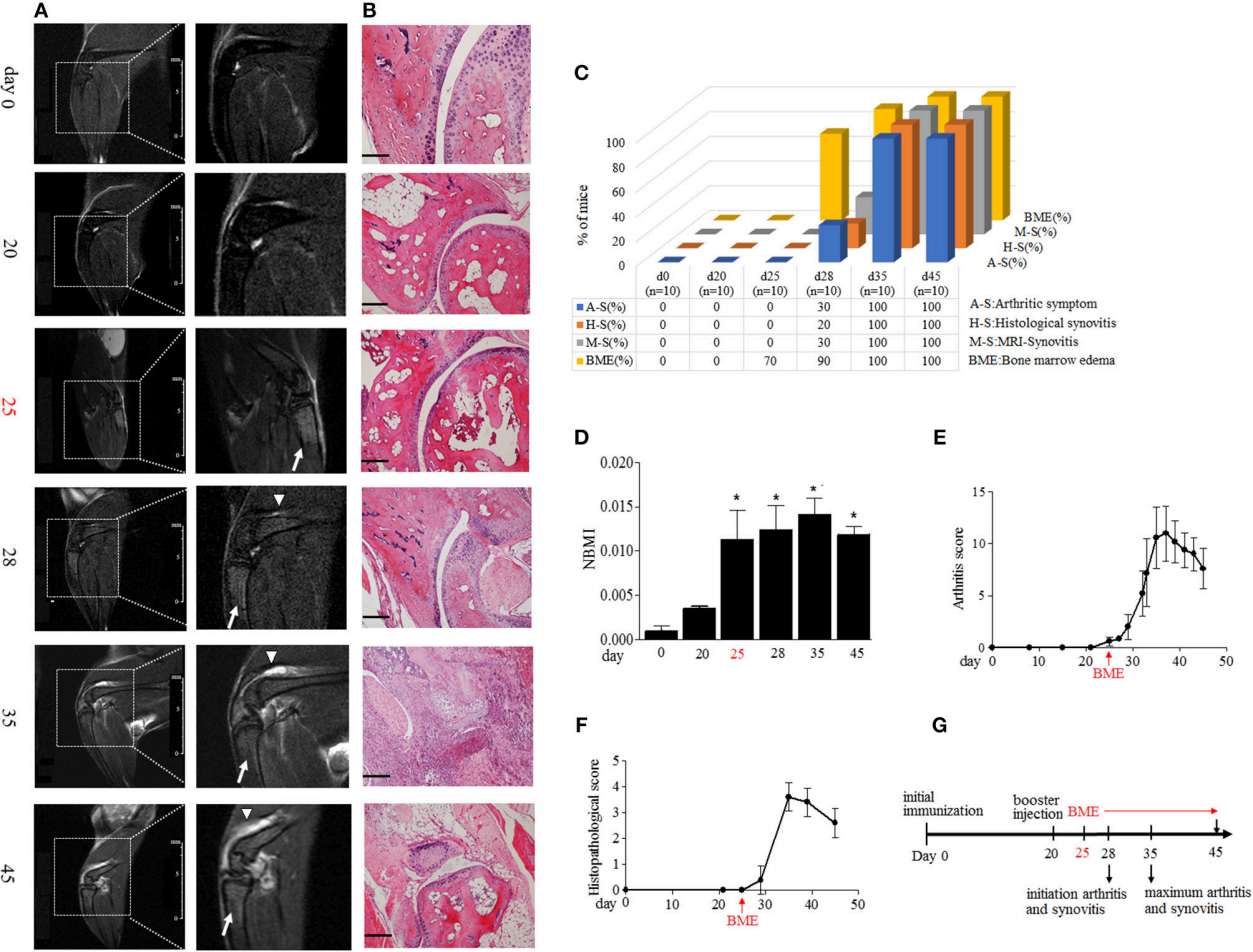
BME precede the arthritic symptoms and synovitis during the development of CIA. **(A)** Representative MRI image of knee joints at different time points after first immunization in CIA mice. Insets are enlarged images of knee joints. Arrows indicate short time inversion recovery MRI BME signal. Triangles indicate synovitis. **(B)** Representative histological sections of CIA. Scale bar, 100μm. **(C)** Proportion of the occurrence of arthritic symptoms (A-S), histological synovitis (H-S), MRI detected synovitis (M-S), and BME in mice at the indicated time of CIA process (*n* = 10, at each time point). **(D)** BME in panel A was quantified using normalized bone marrow intensity (NBMI). Values are means ± SD (*p < 0.05, compared with day 0). **(E,F)** Arthritis **(E)** and histopathological **(D)** scores of CIA mice. **(G)** Schematic diagram of experiments and the time point of appearance of arthritic symptoms, BME, and synovitis during the development of CIA. The first time of BME appearance during the CIA process was marked with red.

Immunization with collagen and CFA induced clinical arthritis with 100% in our DBA/1J mice. We compared the time course of BME emergence and corresponding arthritic symptoms and synovitis during the development of CIA. The BME on the MRI image was defined as increased signal intensity on T2-weighted images in marrow tissues (17). MRI were evaluated by 3 blinded, independent radiologists. As shown in Figure 1, MRI detected that BME could appear as early as at day 25 in 70% mice after the first immunization (Figures 1A,C), when there were not any arthritic symptoms (Figures 1C,E) or histopathological changes (Figures 1B,C,F) in these mice (*n* = 10). At day 28, BME have occurred in 90% mice, whereas the arthritic symptom and histological synovitis only began to be presented in 30 and 20% CIA mice at that time (*n* = 10) (Figure 1C). Mice then underwent a rapid clinical progression and with severe synovitis at around day 35 (*n* = 10) (Figures 1E,F). Accordingly, BME reached the peak level at that time (Figures 1D). In our data, three mice did not show BME signal in MRI at day 25, and the delayed appearance of BME was found at day 28. Interestingly, these three mice displayed the delayed arthritis onset and relative mild symptoms accordingly, as compared with those mice carrying BME at day 25 (data not shown).

MRI has been suggested as a sensitive tool for detecting synovitis in early RA (18). We also compared the time course of the occurrence of BME and synovitis detected by MRI in a same joint in our CIA mice. As shown in Figures 1A,C, a weak synovitis signal could only be detected in three mice (30%) at day 28; conversely, about 70% mice have shown the robust BME signal at day 25.

Taken together, as summarized in Figure 1G, these data suggest that the appearance of BME antedate clinical arthritis, histological, and MRI synovitis during the development of CIA.

### BME Links to the Increased Osteoclast Formation in Bone Marrow During the Development of CIA

**Figure 2:**
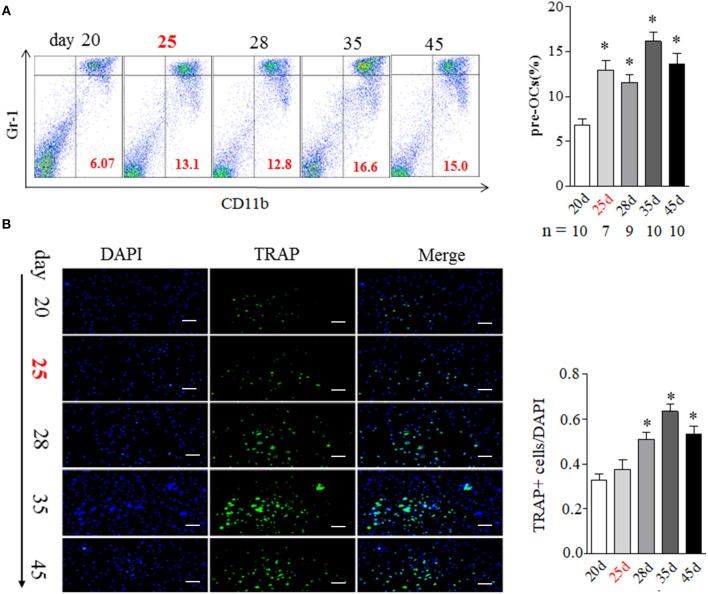
BME links to the increased osteoclast formation in bone marrow during the development of CIA. **(A)** CD11b^+^Gr-1^low^ OCs precursors in bone marrow detected by flow cytometry. Graph show the quantitation data (sample size used in this experiment was indicated under the Graph). **(B)** TRAP positive cell in bone marrow detected by immunofluorescence staining (*n* = 5). Values are means ± SD (*p < 0.05, compared with day 20). All experiments were repeated three times. Scale bar, 100 μm.

BME has been suggested as the strongest predictor of bone erosion in RA patients. Though the precise mechanism remains elusive, osteoclasts (OCs) are the cells ultimately responsible for bone destruction in RA. OCs are formed from bone marrow hematopoietic precursors. We addressed the correlation of BME with OCs in bone marrow during the development of CIA. As shown in Figure 2A, the proportion of OCs precursors, as detected by CD11b^+^Gr-1^low^ expression using flow cytometry, were significantly increased in bone marrow cells from day 25 mice with BME *(n* = 7), comparing with day 20 mice without BME (*p* < 0.05) (Figure 2A). We also analyzed the changes in the composition of bone marrow cells using different myeloid markers including Ly6C and F4/80 and the similar increased trend was been found in these markers from day 25 mice with BME (Figure S1). The OCs precursors numbers in bone marrow cells from three mice without BME at day 25 were also analyzed. As expected, the proportion of OCs precursors is higher in mice with BME than those without BME (Figure S2). Immunofluorescence staining were further performed on isolated bone marrow cells that have been cultured for 5d to confirm the OCs expression in CIA mice. As expected, the concomitant increased expression of TRAP+ cells were found in bone marrow cells from CIA mice after day 28 (*n* = 5) (Figure 2B). These data have suggested that BME is associated the increased OCs number in bone marrow during the CIA process.

**Figure 3:**
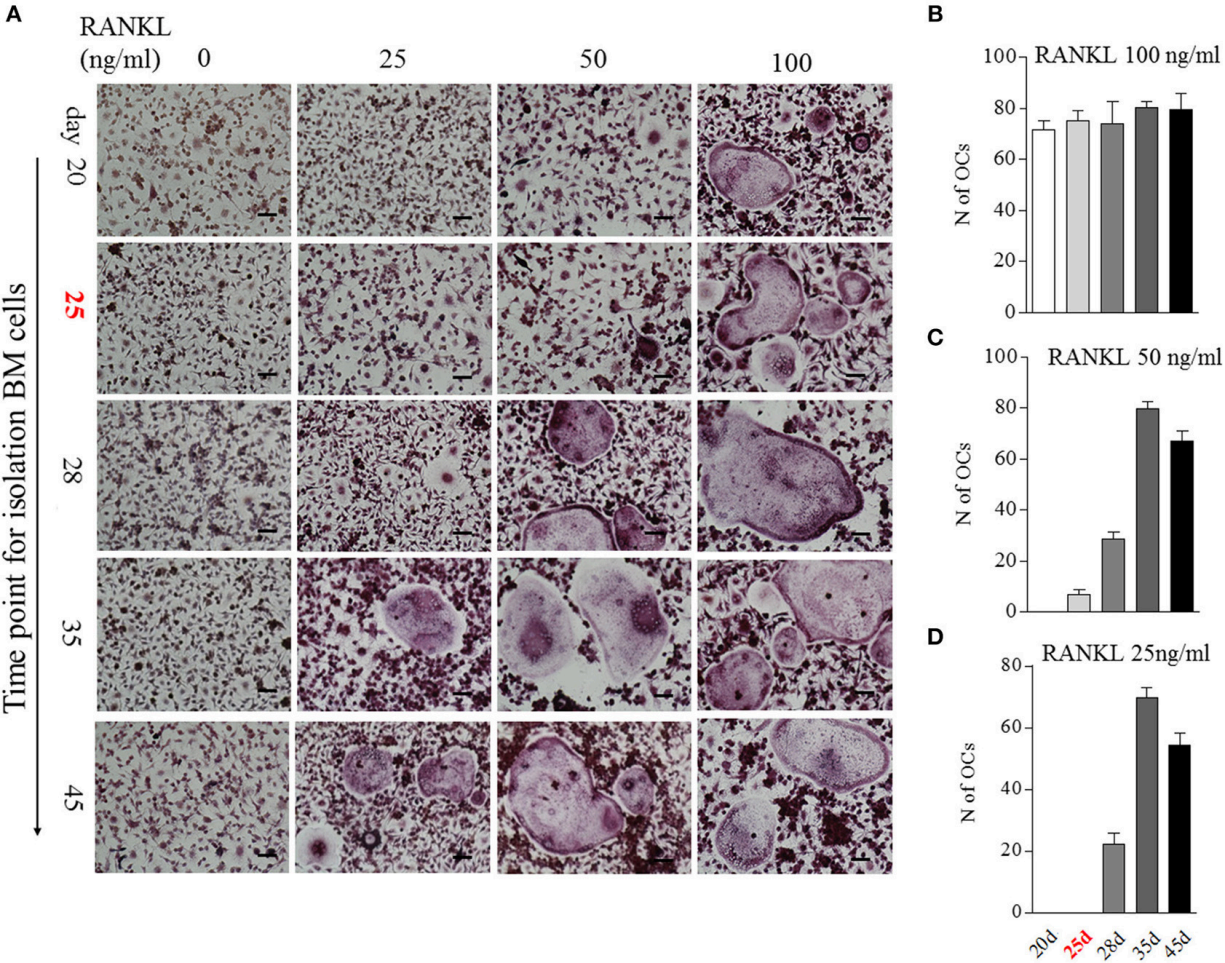
The impact of BME on the differentiation bone marrow cells into OCs *in vitro*. **(A)** Bone marrow cells from different time points of CIA process were treated with C-MSF for 2 days and then were stimulated with a series of concentrations RANKL (0, 25, 50, and 100 ng/ml) for 5d. The OCs formation was assayed by TRAP staining. **(B–D)** Graphs show the quantitation data of the effect of different dose of RANKL on the OCs formation in bone marrow cells from indicated time of CIA (*n* = 5). All experiments were repeated three times. Scale bar, 100 μm.

We then analyzed the impact of BME on OCs formation using *in vitro* assays. Bone marrow cells isolated from different time points of CIA process were used to induce osteoclastogenesis. Our previous study has indicated that 100 ng/ml RANKL is an ideal dose for inducing OCs formation (16). In current experiment, after being treated with M-CSF for 2 days, these bone marrow cells were stimulated with a series of concentrations RANKL (0, 25, 50, and 100 ng/ml, respectively) for 5 days. As expected, a full dose of 100 ng/ml RANKL was required for inducing OCs formation in bone marrow cells from day 20 CIA mice after first immunization (Figures 3A,B). Bone marrow cells from day 25 CIA mice with BME could generate OCs under half dose of RANKL (50 ng/ml) stimulation (Figures 3A,C). Moreover, only a quarter dose of RANKL (25 ng/ml) was required for inducing OCs formation in bone marrow cells from day 28 (Figures 3A,D). Taken together, these data demonstrate that BME links to the increased osteoclast formation in bone marrow during the development of CIA. Moreover, our data imply that bone marrow cells derived from BME period CIA might possess some endogenous signals, making it for a minor RANKL sufficient to drive osteoclastogenesis.

### BME Links to the Increased OCs in Marrow Sides of the Subchondral Bone Surface During the Development of CIA

**Figure 4:**
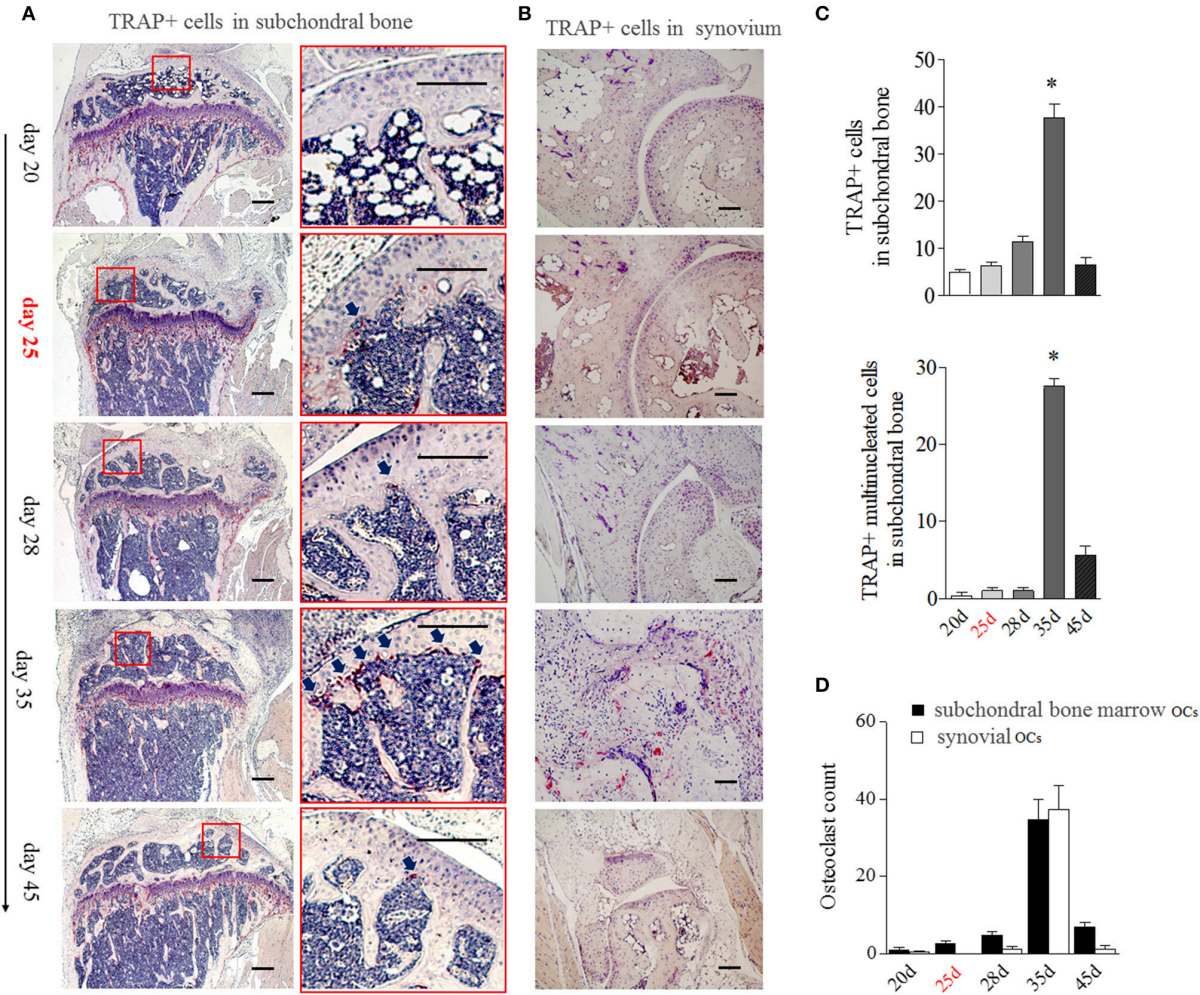
BME links to the aggregated OCs in bone marrow adherent to the subchondral bone surface. **(A)** Representative image of TRAP positive cells in subchondral bone (*n* = 5). The region with the solid line indicates areas of interest to be enlarged in the right image. Arrows indicate TRAP positive cells. **(B)** Representative image of TRAP positive cells in synovium (*n* = 5). **(C)** Graphs show the quantitation data of image A. Values are means ± SD (*p < 0.05, compared with day 20). **(D)** The OCs count in subchondral bone marrow and in synovium. The first time of BME appearance during the CIA process was marked with red. All experiments were repeated three times.

Since the appearance of BME is associated with the enhanced OCs formation in bone marrow, we then analyzed the distribution of OCs in the bone marrow by tartrate-resistant acid phosphatase (TRAP) histochemistry. Given TRAP+ multinucleated cells might represent bona fide OCs, we evaluated the changes of total TRAP+ cells and TRAP+ multinucleated cells in bone, respectively. As shown in Figure 4A, TRAP+ cells were mainly distributed along the marrow bone surface and around the growth plate at day 20. However, after appearance of BME (around day 25), the number of TRAP positive cells (Figure 4A above) and TRAP+ multinucleated cells (Figure 4A below) in marrow sides of the subchondral bone surface would tend to rise. At day 35 (peak level of BME), the total TRAP+ cells and TRAP+ multinucleated cells were significant increase in endosteum of subchondral bone as compared with those at day 20 (*p* < 0.05) and then sharply decreased at day 45 (Figures 4A,C).

The obviously TRAP+ cells in synovial tissue only could be detected at around day 35, at that time with the markedly synovitis and synovial hyperplasia in CIA mice (Figure 4B). We compared TRAP+ multinucleated cells number in “inside” (marrow sides of subchondral bone) with “outside” (synovial tissue) during the development of CIA. As shown in Figure 4D, there were more OCs in subchondral bone marrow than those in synovial tissue from day 20 to 28. The inside and outside OCs numbers reached to the similar level at day 35. Our data suggest that BME links to the increased OCs in marrow sides of the subchondral bone surface during the development of CIA.

### BME Links to the Increased Osteoclastic Activity During the Development of CIA

**Figure 5:**
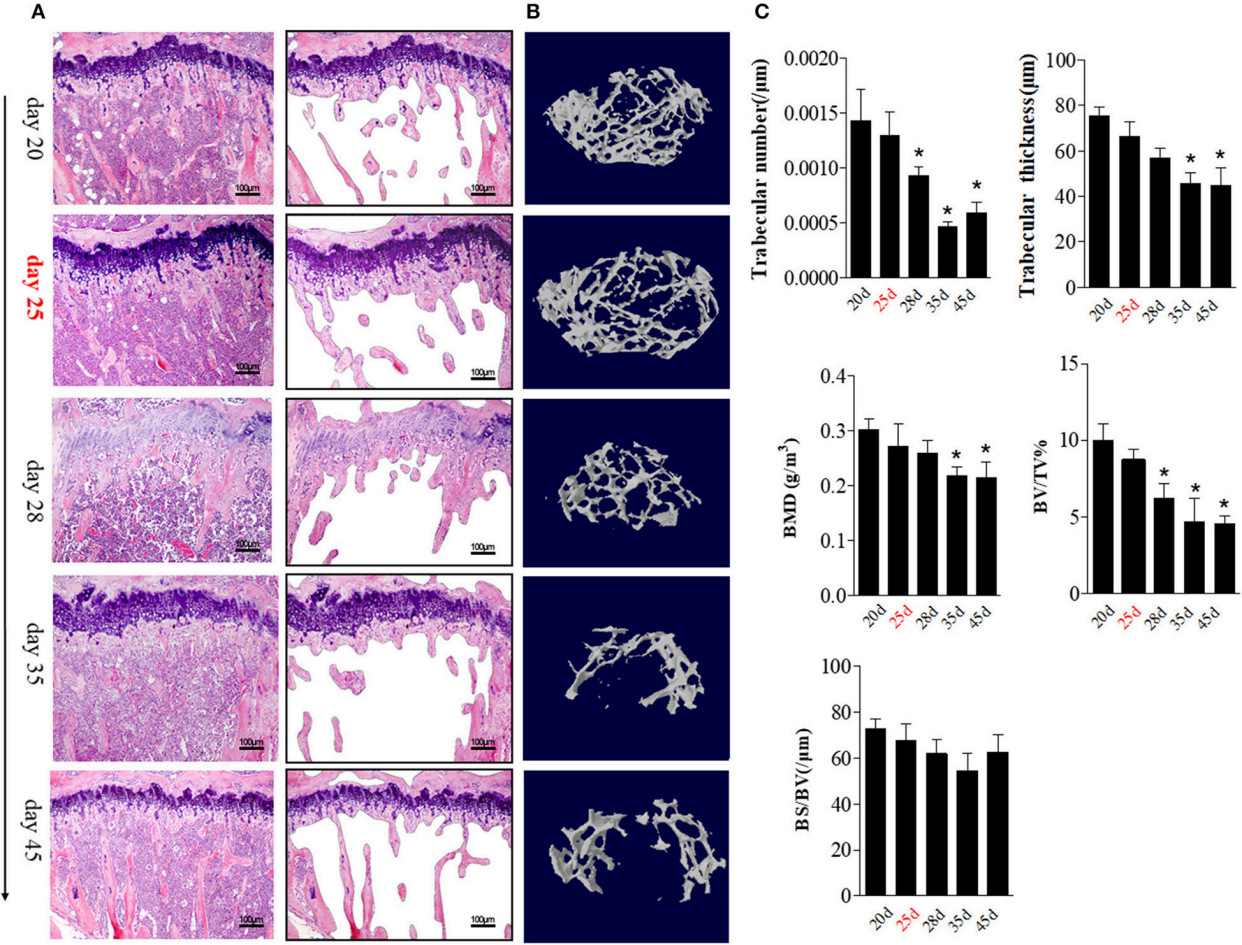
BME links to the changes of trabecular bone during the development of CIA. **(A)** HE staining showed the changes in trabecular bone at the indicated time (*n* = 5). The trabecular bone outline shaped by photoshop software was showed on the right. **(B)** Representative longitudinal 3D micro-CT images showing changes in trabecular bone microarchitecture regions from the CIA mice at the indicated time point. **(C)** Structural parameters in these CIA mice. Values are means ± SD (*p < 0.05, compared with day 20). The first time of BME appearance during the CIA process was marked with red. All experiments were repeated three times.

The aforementioned data suggest that BME is associated with the increased number and altered distribution of OCs in bone marrow. We then explore the potential impacts of these changes on bone erosion. Compared with other time points, an irregular eroded surface was found in subchondral bone from day 35 CIA mice, when there were significant increased OCs aggregation on the endosteal bone surfaces (Figure 4A, right). We further assessed the changes of bone trabecula number during the CIA development using HE staining. At day 25, accompanied by presence of BME and increased OCs precursors in bone marrow, HE staining indicated that the number of trabecular bones have a decreased trend as compared with those at day 20 (Figure 5A).

We quantified the bone microarchitecture values of bone volume, trabecular thickness, trabeculae number, and bone mineral density using Micro-CT. Levels trabeculae number and bone volume was statistically significant decreased from day 28 (as compared with day 20, *p* < 0.05) and reached its lowest level at day 35 (*p* < 0.05).

Trabeculae thickness and bone mineral density was decreased from day 35 CIA mice (Figures 5B,C).

These data support a functional role of BME in osteoclastic activity and local bone loss during the CIA process.

### BME Links to the Changed OCs-related Cytokines and Immune Cells Expression in Bone Marrow During the Development of CIA

**Figure 6:**
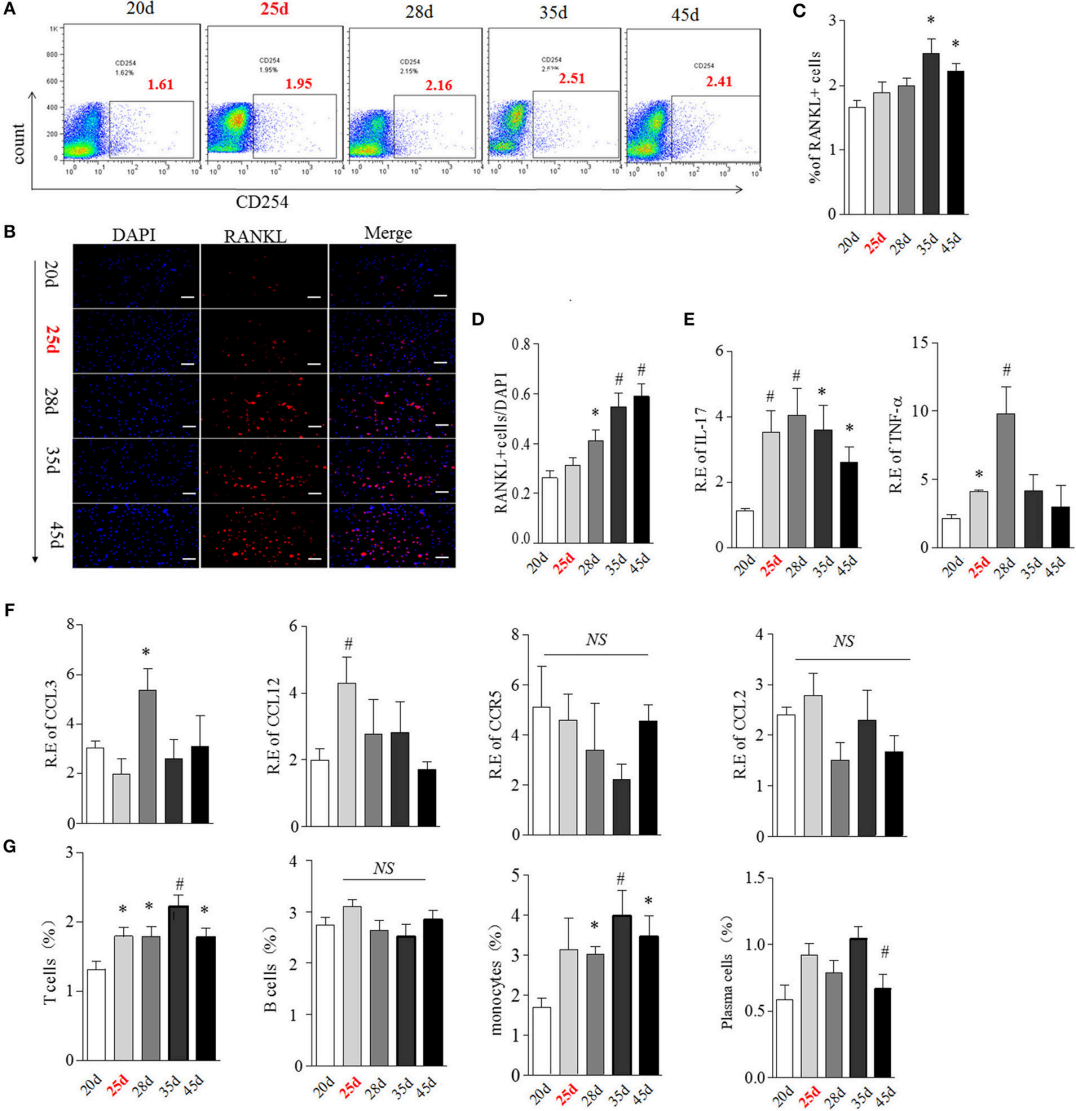
BME links to the increased RANKL, OCs-related cytokines and immune cells expression in bone marrow during the development of CIA. **(A,B)** Expression of RANKL in bone marrow cells detected by flow cytometry **(A)** and immunofluorescence staining **(B)**. **(C,D)** Graphs show the quantitation data from image A **(C)** and B **(D)**. **(E)** Expression of IL-17(left) and TNFα(right) in bone marrow cells detected by Real-time PCR. **(F)** Expression of CCL3, CCL4, CCL12, and CCR5 in bone marrow cells detected by Real-time PCR. **(G)** Expression of T cell, B cell, plasma cell and monocyte in bone marrow cells were detected by flow cytometry. *n* = 5 at each indicated time point. Values are means ± SD (*p < 0.05, #*p* < 0.01, compared with day 20). All experiments were repeated three times.

We then hypothesized that bone marrow might create a unique “osteoclastic environment” accompanied by BME in the disease course, facilitating OCs formation and function. RANKL plays a pivotal role in the differentiation, function and survival of osteoclasts (19). We first tested the time-course changes of RANKL expression in bone marrow cells from CIA. We previously reported that the obvious RANKL expression could be detected in joint tissue at around day 35 CIA mice (16). Here, RANKL positive cells showed an increase trend in bone marrow cells from day 28 to 35 and reach the statistical differences at day 35 (*p* < 0.05, as compared with day 20) (Figures 6A,C). The similar increased trend of RANKL positive cells were confirmed in isolated bone marrow cells by immunofluorescence staining, which reached the statistical differences from day 28 (*p* < 0.05, as compared with day 20) (Figures 6B,D).

It has been suggested that multiple cytokines and chemokines contribute to driving OCs differentiation and migration (19). We next examined the transcription of several OCs-related pro-inflammatory cytokines and chemokines, including IL-17, TNFα, CCL3, CCL4, CCL12, CCR5 by Real-time PCR. IL-17 mRNA was elevated from day 25, reaching a peak level at day 28 (*p* < 0.01, respectively, as compared with day 20) and thereafter gradually decreasing (Figure 6E, left). TNFα was also up-regulated from day 25 (*p* < 0.05, as compared with day 20) and reached a peak level at day 28 (*p* < 0.01, as compared with day 20), and sharply decreased after day 28 (Figure 6E, right). Expression of CCL3 and CCL12 were higher merely at day 28 (*p* < 0.05) and day 25 (*p* < 0.01), while there was no significant change in CCR5 and CCL4 mRNA expression in bone marrow in the CIA process (Figure 6F).

OCs, derived from the monocyte/macrophage lineage, lived with immune cells together in a same bone marrow. The cross-talk between OCs and immune cells played a critical role in in bone remodeling (20). Next, we characterized the expression of T cell (CD4), B cell (B220), plasma cell (CD138), and monocyte (CD14) during the development of CIA using Flow cytometry analysis. As shown in Figure 6G, the proportion of T cells was significantly higher from day 25 to 45, reaching the peak level at day 35 (*p* < 0.01, as compared with day 20). Consistent with T cells, the proportion of monocytes was also markedly elevated from day 25 to 45 (*p* < 0.05 at day 28 and 45, *p* < 0.01 at day 35, as compared with day 20). No differences in B cells and plasma cells were observed at any time points during CIA process.

RANKL could be expressed on active T cell, B cell, plasma cell and monocyte (21, 22). We next analyzed the cellular sources of RANKL in bone marrow by comparing expression of RANKL on these immune cells during CIA process using flow cytometry. Our data indicated that RANKL was mainly high expressed on bone marrow T cell and monocyte from day 25 to 45 CIA mice (Figure S3).

Taken together, these data suggested that the appearance of BME links to a changed bone marrow microenvironment, comprising of high expressed RANKL, various OCs-related cytokine and activated T cells and monocytes.

## Discussion

RA is conventionally considered as an archetypal disease of the synovial tissue (23). Because clinically detected synovitis precedes radiographic bone erosion, it is commonly thought the disease starts in the synovia and then later invades into the bones. Since the term “bone marrow edema” (BME) was first described by Wilson and colleagues on MRI images in 1988 (24), the concept of “BME” has become integrated into our understanding of pathogenic mechanisms in RA. Robust evidences have suggested that BME is a strong predictor of progression of erosion in RA. Here, we use a CIA model to study these parameters in a same joint longitudinally and provide the evidence that BME reflects a unique marrow “osteoclastic environment,” prompting OCs formation and function.

In the traditional paradigm, the “outside-inside” mechanism is the main cause to driving bone erosion (23). In this model, BME occurs secondary to synovial pathobiological signals. In our CIA model, we found that the 70% BME presented at approximately day 25 after first immunization, preceding the arthritic symptoms and histological or MRI synovitis during the development of CIA. BME has been suggested as an early event in RA process. Our data provide the direct evidence that the pathological changes in bone marrow might antedate the changes in synovium in CIA mice, highlighting the important role of bone marrow signals in RA development.

The authors describe that most OCs were seen at d 35 the peak level of BME. However, it should be noted that also synovitis and joint swelling had their peaks at d 35. This should be discussed in the manuscript.

How BME contributes to RA pathogenesis remains unclear. According to the popular hypothesis of “two-hit model” for RA pathogenesis (1), the “first hit” breaks immune tolerance resulting in T, B cell activation and autoantibody production; the “second hit” leads to amplification of inflammation in synovium. Bone marrow is consisting of hematopoetic cells, immune cells and bone cells. The emerging field of osteoimmunology has proved that bone marrow is a crucial part of the immune system (20). We found that accompanying the appearance of BME, the numbers of T cell, monocyte and inflammatory cytokine were significantly increased in bone marrow. These data imply that bone marrow might be a critical pathologic site for “first hit” to break immune tolerance in RA. It is possible that under the interaction of genetic and environmental factors, certain antigens activate an adaptive immune response in bone marrow or other lymphoid tissue triggering the “first hit” and then trigger synovial inflammation.

An important novel finding of the present study is that the appearance of BME is linked to a unique bone marrow “osteoclastic environment” during the development of CIA. The bone marrow has been proved to consist of “niche” in controlling quiescence, proliferation, differentiation and self-renewal capacity of hematopoietic stem cell (25). OCs originate from bone marrow hematopoietic precursors. Undoubtedly, bone marrow microenvironment signal participates in regulating OCs formation and function (19). Whether there is a similar OCs -related “niche” remains unclear.

BME is correlated with a highly cellular infiltrate within the bone marrow in our data, as supported by bone marrow biopsy data from human RA patients (23, 26, 27). However, these human RA studies only could provide the cross-sectional data. By observing CIA development longitudinally, we have found that after the appearance of BME, bone marrow creates a critical regulatory milieu that prompts OCs formation and function. The sparing RANKL used in the *in vitro* induction study provides the direct evidence that bone marrow cells from BME period carries the endogenous osteoclastogenesis signals. We identified that the BME-linked environment is consisting of a remarkable increased RANKL expression and up-regulated various types of OCs-related immune cells and proinflammatory cytokines. Our data could explain the strong link between BME and bone erosion in RA.

The most OCs were seen in bone marrow at day 35 coinciding with the peak level of BME, synovitis, and joint swelling. The enhanced OCs-related immune cells and proinflammatory cytokines expression in the bone marrow might prompt OCs generation accompanied by the disease progression. Previous study showed that synovial hyperplasia could erode bones and then create bone canals that link the bone marrow and the synovium in CIA (28). Although no obvious bone canals were observed it in our CIA model, the evident cortical bone broken have been found in our data. More interestingly, our study revealed an irregular eroded surface in subchondral bone from day 35 CIA mice, suggesting that an “inside to outside” bone resorption process was working here. It is possible that these aggregate OCs could erode subchondral bone surface, contributing to the bone canals formation. Thus, we hypothesis that bone marrow “osteoclastic environment” might stem from the abnormal immuno-inflammatory response in bone marrow, and synovitis could prompt inflammation process in bone marrow by bone marrow-synovial connection or by circulation.

In conclusion, our data demonstrate that BME is linked to a unique marrow “osteoclastic environment” and that precedes the synovitis and arthritic symptoms during the development of CIA. These results emphasize the importance of the bone marrow microenvironment on bone erosions in RA and advocate that the “treat to target” strategy should take into account BME.

## Ethics Statement

The animal experiments were performed in accordance with the guidelines approved by Institutional Animal Care and Use Committee of Nanjing Medical University. The protocol was approved by the Institutional Animal Care and Use Committee of Nanjing Medical University.

## Author Contributions

WT conceived, designed, and performed the majority of the described studies and was responsible for initial versions of this manuscript. AL, FW, WX, and KG undertook the acquisition and analysis of data. FW, WX, QW, QZ, MZ, LQ, and WT contributed to the analysis and interpretation of the data. All authors contributed to the drafting and read and approved the manuscript.

### Conflict of Interest Statement

The authors declare that the research was conducted in the absence of any commercial or financial relationships that could be construed as a potential conflict of interest.

## Supplementary Material

Figure S1 The changes in the composition of bone marrow detected different myeloid markers during indicated time point of CIA development. A. Ly6C and B. F4/80.

Figure S2 CD11b^+^Gr-1low OCs precursors in bone marrow at day 25 CIA mice with (n = 7) or without BME (n = 3) detected by flow cytometry. Graph show the quantitation data.

Figure S3 The expression of RANKL on T cell, B cell, plasma cell and monocyte in bone marrow during CIA process using flow cytometry. Graph show the quantitation data. ^*^*p*<0.05, #*p*< 0.01, as compared with day 20.

Table S1 PCR primer.

## Abbreviations

BME: bone marrow edema
CIA: collagen-Induced arthritis
FLS: fibroblast-like synoviocytes
OCs: osteoclasts
MRI: Magnetic resonance imaging
M-CSF: macrophage colony-stimulating factor
RA: rheumatoid arthritis
RANKL: nuclear factor κB ligand
TRAP: tartrate-resistant acid phosphatase.
