# Supplementary figures and images for: The Bone Marrow Edema Links to an Osteoclastic Environment and Precedes Synovitis During the Development of Collagen Induced Arthritis
Source: Front Immunol. 2019 Apr 24;10:884. doi: 10.3389/fimmu.2019.00884 (PMC6491763; doi:10.3389/fimmu.2019.00884)

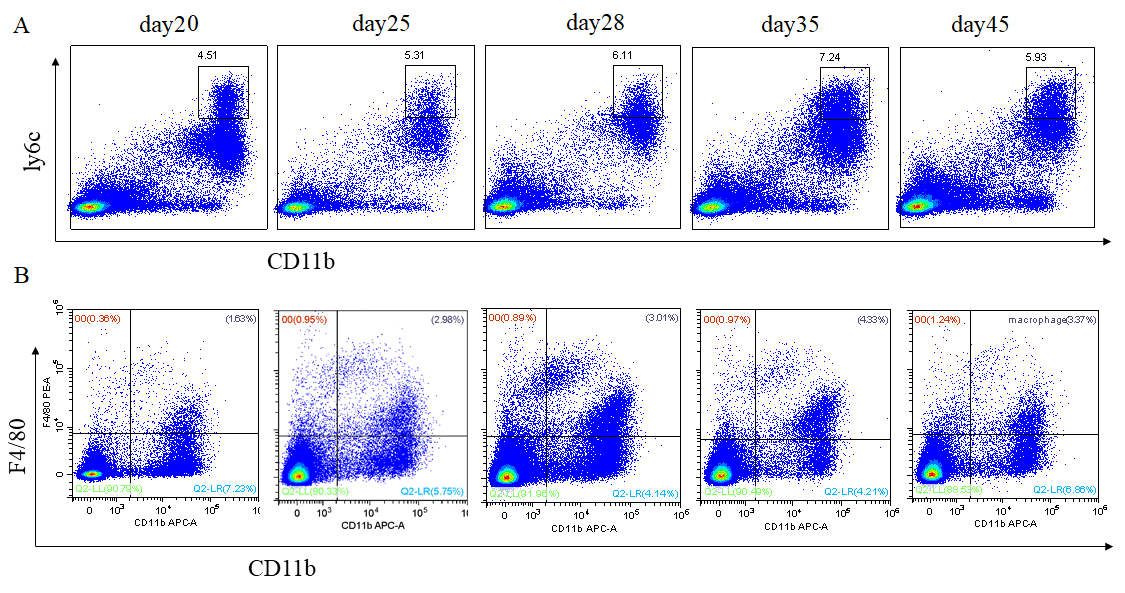

Supplement: Figure S1 — The changes in the composition of bone marrow detected different myeloid markers during indicated time point of CIA development. A. Ly6C and B. F4/80. [file Image_1.tif]

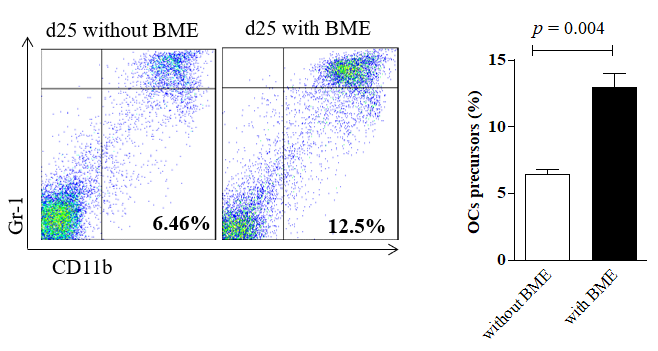

Supplement: Figure S2 — CD11b+Gr-1low OCs precursors in bone marrow at day 25 CIA mice with (n = 7) or without BME (n = 3) detected by flow cytometry. Graph show the quantitation data. [file Image_2.tif]

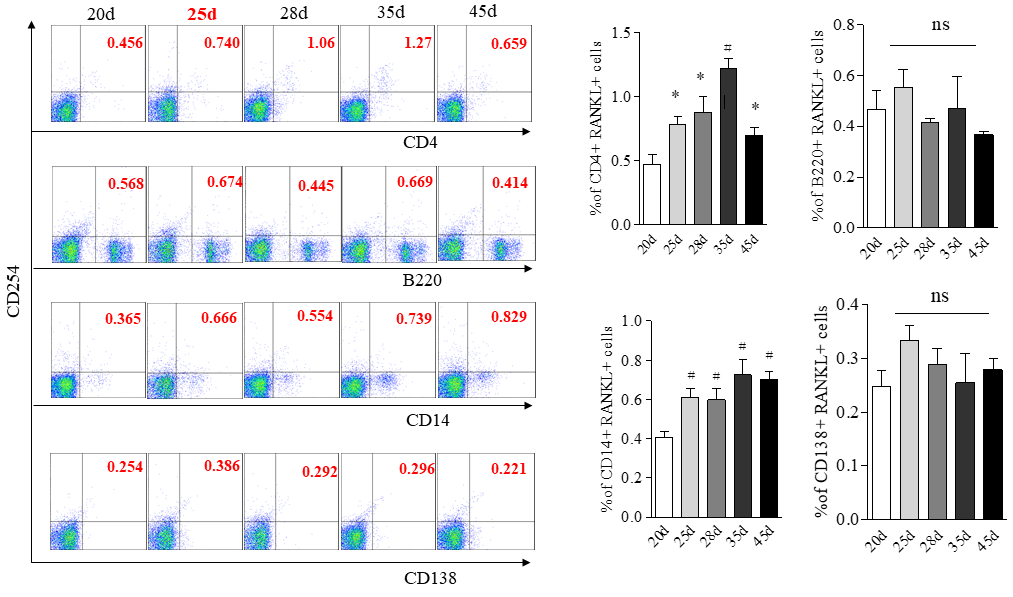

Supplement: Figure S3 — The expression of RANKL on T cell, B cell, plasma cell and monocyte in bone marrow during CIA process using flow cytometry. Graph show the quantitation data. *p<0.05, #p< 0.01, as compared with day 20. [file Image_3.tif]
