# Supplementary material for: The Bone Marrow Edema Links to an Osteoclastic Environment and Precedes Synovitis During the Development of Collagen Induced Arthritis
Source: Front Immunol. 2019 Apr 24;10:884. doi: 10.3389/fimmu.2019.00884 (PMC6491763; doi:10.3389/fimmu.2019.00884)
Supplement: Table S1 — PCR primer. [file Table_1.DOCX]

Table S1. PCR primer

| Genes | Forward | Reverse |
| --- | --- | --- |
| CCL2 | TTCACTGGCAAGATGATCCC | TGCTTGAGGTGGTTGTGGAA |
| CCL3 | ATGCAGGTCTCCACTGCTGC | TCAGGCACTCAGCTCCAGGTC |
| CCL12 | CCGGGAAGCTGTGATCTTCA | GACTTCTGATCCAAGTGGTTCATG |
| CCR5 | GATAGGTACCTGGCTGTCGTCCAT | ACCAGCCCCAAGATGACTATCT |
| TNFα | CTGCCCCAATCCCTTTATT | CCCAATTCTCTTTTTTGAGCC |
| IL17 | TTTAACTCCCTTGGCGCAAAA | CTTTCCCTCCGCATTGACAC |
| GAPDH | TGCACCACCAACTGCTTAGC | GGCATGGACTGTGGTCATGAG. |
